# Overview of Functionalized Porous Materials for Rare-Earth Element Separation and Recovery

**DOI:** 10.3390/molecules29122824

**Published:** 2024-06-13

**Authors:** Yong Peng, Pingxin Zhu, Yin Zou, Qingyi Gao, Shaohui Xiong, Binjun Liang, Bin Xiao

**Affiliations:** 1 Key Laboratory of Mine Geological Disaster Prevention and Control and Ecological Restoration, School of Resources and Civil Engineering, Gannan University of Science and Technology, Ganzhou 341000, China; 18870241904@163.com (Y.P.); 13755740765@163.com (P.Z.); 15978371476@163.com (Y.Z.); gqy15757376485@163.com (Q.G.); liangbinjun1205@163.com (B.L.); 2 Hunan Provincial Key Laboratory of Advanced Materials for New Energy Storage and Conversion, School of Materials Science and Engineering, Hunan University of Science and Technology, Xiangtan 411201, China; 3 Key Laboratory of Ionic Rare Earth Resources and Environment, Ministry of Natural Resources of the People’s Republic of China, Jiangxi College of Applied Technology, Ganzhou 341000, China

**Keywords:** porous materials, adsorption method, rare-earth elements, separation and recovery, research status

## Abstract

The exceptional photoelectromagnetic characteristics of rare-earth elements contribute significantly to their indispensable position in the high-tech industry. The exponential expansion of the demand for high-purity rare earth and related compounds can be attributed to the swift advancement of contemporary technology. Nevertheless, rare-earth elements are finite and limited resources, and their excessive mining unavoidably results in resource depletion and environmental degradation. Hence, it is crucial to establish a highly effective approach for the extraction and reclamation of rare-earth elements. Adsorption is regarded as a promising technique for the recovery of rare-earth elements owing to its simplicity, environmentally friendly nature, and cost-effectiveness. The efficacy of adsorption is contingent upon the performance characteristics of the adsorbent material. Presently, there is a prevalent utilization of porous adsorbent materials with substantial specific surface areas and plentiful surface functional groups in the realm of selectively separating and recovering rare-earth elements. This paper presents a thorough examination of porous inorganic carbon materials, porous inorganic silicon materials, porous organic polymers, and metal–organic framework materials. The adsorption performance and processes for rare-earth elements are the focal points of discussion about these materials. Furthermore, this work investigates the potential applications of porous materials in the domain of the adsorption of rare-earth elements.

## 1. Introduction

Since 2010, rare-earth elements (REEs) have been classified as strategic resources by the European Union and constitute crucial components of high-tech and everyday consumer goods [1]. Rare-earth elements, distinguished by their unique 4f electron structure, exhibit excellent optical, electrical, and magnetic properties. Due to their ability to form high-performance functional materials in combination with other elements, rare-earth elements serve as the core components in a plethora of novel functional materials [2]. Various REE-containing functional materials, such as rare-earth magnetic materials, hydrogen storage materials, luminescent materials, catalytic materials, etc., find widespread applications in metallurgical machinery [3], petrochemicals [4], light industry and agriculture [5], energy and environmental protection [6], defense and military industries [7], and high-tech materials [8]. For instance, in the medical field, Chao Mi et al. [9] have introduced a novel, non-radiative, high-resolution method for diagnosing skeletal diseases using lanthanide-doped nanocrystals in high-resolution near-infrared II (NIR-II) imaging. The application prospects of rare-earth elements in high-tech fields are extensive, making them indispensable strategic materials for the development of advanced technologies and cutting-edge defense technologies [10].

**Figure 1:**
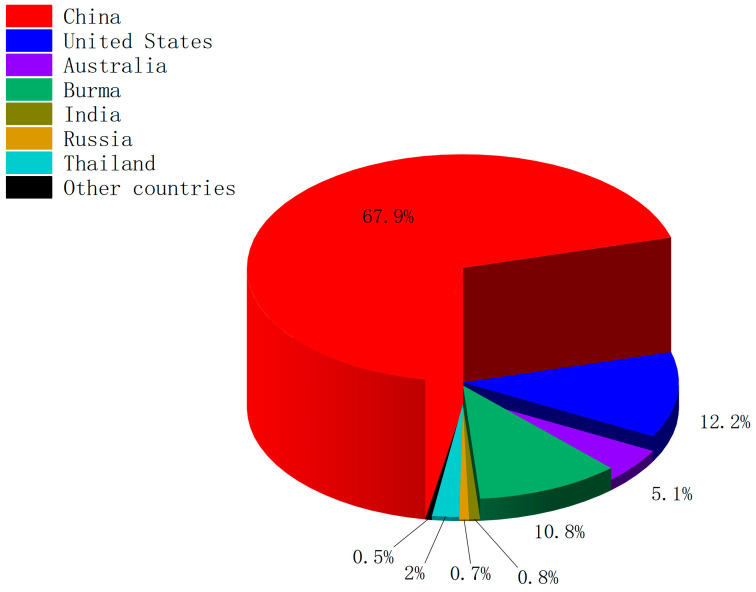
Global mine production of rare-earth oxides in 2023.

Due to the rapid growth in the demand for rare earths, the global production of rare-earth oxides (REOs) continues to rise. There are global variations in the production, consumption, and processing of rare-earth element resources. As shown in Figure 1, in 2023, out of the total global REO production of 350,000 tons, China dominated with 240,000 tons, followed by the United States and Burma [11]. China also holds a leading position in global REO processing and is the largest consumer of rare-earth elements [12]. The large-scale extraction of rare-earth elements (REEs) can significantly damage environmental ecosystems, posing a threat to the health of local residents through contaminated soil and water sources [13]. In addition, research indicates that rare earth mining can also increase antibiotic resistance in the surrounding soil [14]. Currently, despite the growing demand for rare-earth materials, only about 1% of the rare-earth elements in waste containing REEs are recycled [15]. Therefore, finding efficient methods for recycling rare earths has attracted the attention of many researchers who aim to reduce the need for extensive REE mining.

Extraction and adsorption are effective methods for rare earth recovery. Existing rare earth separation technologies include ion exchange, solvent extraction, chemical precipitation, adsorption [16], and membrane separation [17]. Among these, adsorption is considered an effective technique for rare earth recovery. Compared to other separation methods, adsorption technology offers several significant advantages, such as minimal waste, the reduced use of organic solvents, high enrichment factors, and rapid phase separation [18].

This paper reviews the research progress on the adsorption of rare-earth elements using porous materials, including carbon materials, silicon-based materials, porous organic polymers, and metal–organic framework materials. By summarizing and analyzing the relevant literature, the current applications of these materials in the adsorption of rare-earth elements are outlined, and prospects for their future applications in the field of rare-earth element adsorption are discussed.

## 2. Adsorption of Rare-Earth Elements on Porous Materials

Adsorption materials including carbon-based, silicon-based, porous organic polymers, metal–organic framework, and other layered inorganic materials are frequently employed in the recovery of rare-earth elements from mine leachates [19,20,21,22]. Ion exchange is the basis for rare earth adsorption onto these materials. However, due to inadequate adsorption selectivity and recovery efficiency for such complex combinations, ion exchange techniques are severely constrained in their ability to extract rare-earth elements from acidic mine drainage and electronic trash [18]. Functionalized adsorbent materials having interactions unique to rare-earth elements are necessary for the successful selective extraction of rare-earth elements from metal ion combinations.

### 2.1. Carbon-Based Porous Materials

Carbon-based inorganic porous materials, including activated carbon (AC), graphene, graphene oxide (GO), carbon nanotubes (CNTs), carbon nanofibers, carbon quantum dots, and others, have been gaining significant interest. Carbon nanomaterials (CNMs) have an impressive surface area of around 3600 m^2^/g and demonstrate excellent stability in both acidic and alkaline conditions. Carbon-based nanomaterials (CNMs) possess diverse oxygen-containing functional groups, including hydroxyl, carbonyl, and carboxyl groups, which have the ability to bind rare-earth element (REE) ions. The functional groups present in carbon nanomaterials (CNMs) can be further altered and fixed in place to facilitate specific and selective interactions with ligands. The many surface functional groups, in conjunction with the interactions between these groups and rare-earth metal ions, greatly augment the adsorption capability of carbon-based materials. The significant surface area is well acknowledged to possess considerable promise as an adsorbent.

#### 2.1.1. Activated Carbon (AC)

Activated carbon (AC) is a carbon material that can be produced through the pyrolysis and activation of organic substances such as wood, coal, and petroleum coke. Possessing a well-developed pore structure, large specific surface area, and abundant surface chemical groups, AC exhibits a high capacity for specific adsorption.

Selective adsorption of certain metals by AC has been explored, particularly in the recovery of heavy metals like La(III) from highly diluted electrolytes [23]. However, AC’s inherent lack of strong selective metal adsorption can be addressed through various modification methods. The introduction of different functional groups enhances the chemical properties of carbon-based compounds, enabling selective metal adsorption. For instance, treating AC with triphenylphosphine resulted in composites capable of recovering up to 344 mg/g of Dy and Nd from systems with a pH greater than 2.0 [24].

Building on the exploration of modification methods, Han et al. prepared a novel magnetic porous biochar (PBC/ZF) for the efficient adsorption of Ce(IV) in water by loading ZnFeO (ZF) onto porous biochar (PBC) using a one-step hydrothermal method [25]. Karolina et al. established the effectiveness of AC in extracting Re(VII) ions from water-based solutions. They applied the hard and soft acids and bases (HSAB) principle to explain the selectivity of the adsorption process, highlighting the affinity between Re and AC [26].

**Figure 2:**
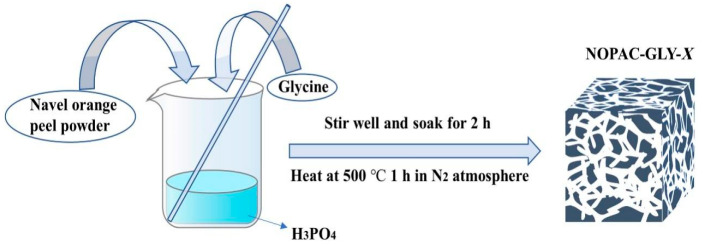
The process of creating activated carbon NOPAC-GLY-X [27].

Activated carbon produced from organic materials demonstrated efficient adsorption of rare-earth elements. As shown in Figure 2, Xiao et al. synthesized NOPAC-GLY-X, an activated carbon adsorbent functionalized with glycine, showing a twofold increase in adsorption capacity for Gd(III) and a remarkable efficiency of 99% at pH 7 [27]. Raphael et al. produced activated carbon from biomass waste and successfully extracted rare-earth elements Ce and La from laboratory-prepared solutions and real leachate [28]. Pinheiro et al. produced environmentally friendly activated carbon (VPW-AC) for adsorbing Ce(III) and La(III) [29].

In further exploration of activated carbon’s versatility, in a study by Burakova et al., activated carbon derived from coconut shells modified with carbon nanotubes exhibited superior adsorption capabilities for Ce and Sc in a sulfuric acid solution [30]. Dong et al. contributed to the field by encapsulating hydroxyapatite- and CaAl-layered double hydroxide in a biochar matrix, creating BC@LDH@HAP with the highest adsorption capacity for Eu(III) [31]. 

Further studies revealed that the introduction of phosphate functional groups on carbon showed better selective adsorption of rare-earth elements. Pei et al. employed a phytate-modified hydrothermal carbonization process to fabricate phytate-assisted sludge hydrochar (P-SHC). Compared to pristine hydrochar, P-SHC exhibits a stronger adsorption capacity for lanthanum, and the research indicates that the enhancement in adsorption performance is primarily attributed to the coordination effect of the phosphate functional groups [32]. Zeng et al. used oil tea husk as raw material to prepare activated carbon via the phosphoric acid activation method, and their phosphoric acid-activated carbon had a high efficiency of Y(III) recovery capacity [33].

Using intermittent and continuous flow adsorption experiments, Joan Serra-Ventura et al. found that biochar made from raw materials such as castor meal, eucalyptus forest residues, bagasse, and coconut shells adsorbed selenium more effectively from aqueous solutions than coal dust and activated carbon [34]. Efthalia Georgiou et al. found that oxidized biochar, produced from palm leaves using HNO_3_, had good adsorption properties for Eu(III) from aqueous solutions [35].

As shown in Table 1, these studies collectively highlight the diverse applications and modifications of activated carbon for the efficient and selective adsorption of various elements. 

#### 2.1.2. Graphene and Graphene Oxide

Graphene is a newly discovered two-dimensional carbon crystal composed of a single layer of carbon atoms. The basic structural unit of fullerenes, carbon nanotubes (CNTs), and graphite is commonly referred to as such. Graphene oxide (GO) is a modified form of graphene that is primarily made up of carbon, oxygen, and hydrogen atoms. Graphene oxide (GO) shares a comparable fundamental structure with graphene, but it also incorporates oxygen-containing functional groups like carboxyl, hydroxyl, epoxide, and others. These functional groups give it good hydrophilicity, enabling interactions with metal ions for the enrichment and separation of metal ions in aqueous phases. Various methods have been developed to enhance the adsorption efficiency of graphene [15].

Moving from the basic characteristics of graphene to practical applications, Manli Li et al.’s experimental findings reveal GO’s significant ability to adsorb La(III) and Nd(II) at rates of 105.5 mg/g and 99.1 mg/g, respectively. Yang X et al. took a step further by producing a hydrogel (GO/P(PNIPAM-MA)) with remarkable stability and adsorption capacity for La(III) ions. This demonstrates how graphene-based materials can be tailored to create advanced adsorbents with specific properties [36]. Ali et al. achieved efficient adsorption of Sm(III) ions using a graphene oxide nanocomposite, highlighting the versatility of GO in adsorbing different rare-earth elements [37]. Similarly, Gao L et al. demonstrated high adsorption capacity for Er(III) ions using a graphene oxide magnetic nanocomposite with immobilized persimmon tannin, further emphasizing the material’s broad applications [38].

**Figure 3:**
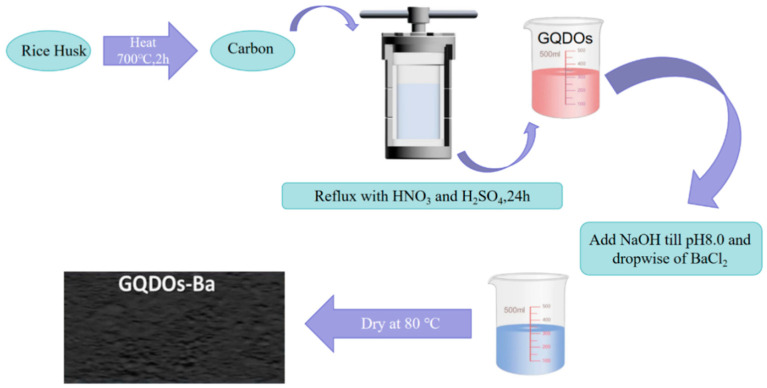
Synthesis of GODOs-Ba nanobiosorbent [39].

Expanding the scope of graphene-based materials, Mahmoud et al. produced graphene quantum dots (GQDOs) from rice husks and created a nanobiocomposite adsorbent with enhanced surface hydroxyl groups. The GQDO production method is shown in Figure 3. This innovative approach demonstrates the potential of utilizing waste materials to synthesize graphene-based adsorbents [39]. Nitrogen-doped nanoscale porous graphene (NDNG) also enters the scene as a promising membrane separation material, selectively engaging in electrostatic interactions with pyrrole-N to effectively separate Sc(III) from other rare-earth ions [40].

Recent research on graphene oxide (GO) has shown its ability to form three-dimensional (3D) structures, as exemplified by the GO-PER composite material [41]. This introduces a new dimension to graphene’s structural versatility. Xu et al. combined corn alcohol-soluble protein (CZ) with GO, resulting in a 3D composite material with excellent mechanical properties for the adsorption of rare-earth elements. This illustrates how the combination of graphene with other materials can lead to the development of advanced adsorbents with enhanced properties [42]. Liao et al. modified GO with 2-amino-benzo-thiazole (ABT), creating a 3D GO-ABT composite aerogel effective in extracting rare-earth elements. The introduction of ABT enhances the material’s adsorption capabilities, showcasing the potential for targeted modifications [43].

Chen et al. synthesized a 3D composite material of graphene oxide-N-benzyloxycarbonyl glycine (GO-ZG) for the efficient adsorption of rare-earth elements. This continues the exploration of 3D graphene-based composites for enhanced adsorption performance [44]. Ren et al. covalently coupled N, N-bis(2-hydroxyethyl) glycine (Bicine) with GO, creating GO-Bicine, which demonstrated excellent selectivity in adsorbing rare-earth elements, phenols, and organic dyes. The covalent coupling of Bicine highlights the importance of chemical modifications for tailoring graphene-based materials to specific adsorption requirements [45].

These studies collectively showcase the diverse applications of graphene-based materials and their modifications, highlighting their potential in adsorption processes for rare-earth elements; see Table 2. From fundamental characteristics to practical applications and innovative modifications, graphene-based materials continue to play a crucial role in advancing adsorption technologies.

#### 2.1.3. Carbon Nanotubes (CNTs)

Carbon nanotubes (CNTs) have shown promise as adsorbents for rare-earth metal ions, but their initial adsorption capacity is constrained. To address this limitation, researchers are actively exploring modifications and oxidations of CNTs, aiming to unravel a more intricate adsorption mechanism. This dynamic research landscape encompasses various studies, each contributing valuable insights to the field.

Khan et al. obtained carboxylated multiwalled carbon nanotubes/poly(o-o-toluidine) nanowires (OH-MWCNT/POT) via microemulsion and in situ oxidative polymerization methods. The nanowires were employed as a cation sensor for the detection of Ce^3+^ in aqueous solution at a modified glassy carbon electrode and were found to exhibit a high sensitivity and ultra-low detection, as well as a good selectivity and a fast response time for Ce^3+^. The experimental results indicate OH-MWCNT/POT nanowire-based sensor has potential applications in the field of environment and health safety [48].

Liu et al. adopted a simple polymerization–cryogenic lyophilization technique to create a three-dimensional network-structured sponge made of carbon nanotubes and chitosan, with imprinted properties (COOH-CNTs/CS-IIS). This innovative material, featuring dispersed imprinting sites, selectively adsorbed Gd(III) ions, emphasizing the potential for selective rare-earth element adsorption [49]. 

**Figure 4:**
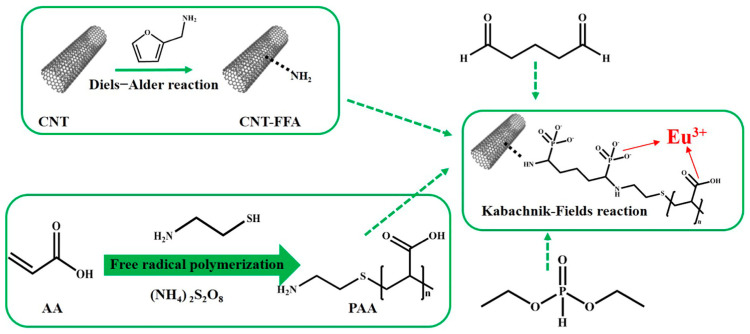
The schematic diagram illustrates the process of creating polymer composites using carbon nanotubes (CNTs) through the DA cycloaddition and KF reactions [50].

Taking a different approach, Guo et al. utilized a Diels–Alder reaction to introduce amino groups to carbon nanotubes through the reaction with furfurylamine. Figure 4 illustrates the production method of the amino-functionalized carbon nanotube composite material CNT-FFA-PAA. A subsequent combination with a polymer, facilitated via the Kabachnik–Fields reaction, resulted in a significant enhancement in its adsorption capacity for Eu(III) ions. This study highlights the versatility of modified CNTs in achieving substantial improvements in adsorption efficiency [50].

In conclusion, while carbon-based nanostructured materials, including activated carbon, graphene oxide, graphene, and carbon nanotubes, exhibit durability in varying conditions, their intrinsic adsorption capacities for rare-earth elements are limited; see Table 3. The ongoing efforts in modifying carbon nanotubes reveal a significant potential for improved adsorption capabilities. Each study contributes a piece to the puzzle, collectively positioning carbon nanomaterials as a highly promising category for rare earth adsorption and extraction. This narrative of continuous improvement underscores the evolving landscape of research in rare earth adsorption technologies.

### 2.2. Silicon-Based Materials

Silica-based mesoporous materials possess a significantly elevated specific surface area and porosity, resulting in enhanced adsorption capacity and contact efficiency. Moreover, the surface of silica-based materials facilitates the attachment or combination of functional groups, thereby playing a crucial role in enhancing the selectivity of adsorbents. Rare-earth ions are commonly Lewis acid ions. Adsorbent materials that have been modified with Lewis base groups such as N, P, -COO, etc., can be advantageous for the separation of rare-earth elements [52].

#### 2.2.1. Silicon-Based Mesoporous Materials Containing Phosphate Groups

**Figure 5:**
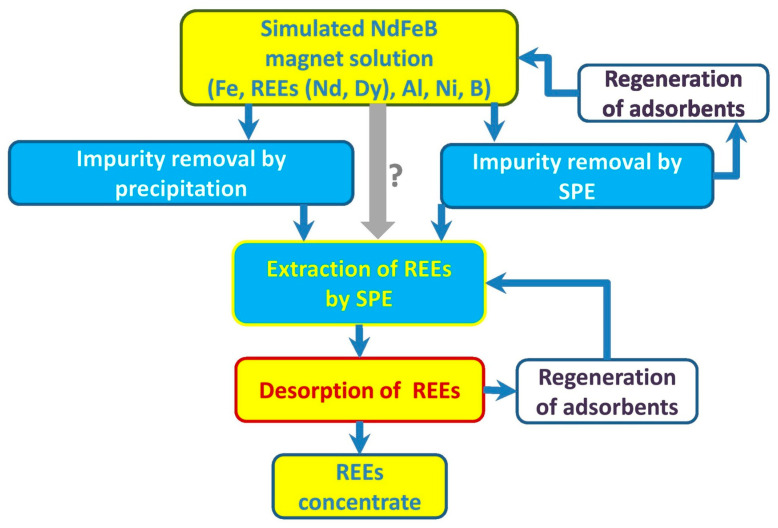
Schematic presentation of REE recovery from simulated NdFeB magnet solution via proposed hydrometallurgical processes [54].

Silica-based mesoporous material adsorbents with phosphate groups have a specific affinity for rare-earth elements, which enables the selective adsorption of REEs. Dudarko et al. synthesized a series of SBA-15-type adsorbents using sodium metasilicate as the main starting reagent using a template method with EDTA, phosphonic acid, and ammonium groups as ligands. The adsorption capacity of the synthesized adsorbents for the target ions was significantly higher than that of pure SBA-15, regardless of the concentration of functional groups [53]. As shown in Figure 5, Dudarko et al. extracted Nd(III) and Dy(III) from simulated solutions using silica functionalized with NH^2−^, EDTA, and phosphonic acid groups. A multistage solid-phase extraction technique using adsorbents functionalized with P/N groups resulted in the complete recovery of rare-earth elements (97.8%) [54].

Boiko et al. sequentially treated silica gel with tetrachlorosilane and conical tetrakisphosphorylated tetrahydroxy (thio) cuproaromatic hydrocarbons to obtain porous organo-organic inorganic adsorbents whose surfaces were covered by spatially ordered phosphonate, hypophosphonate, and phosphine oxide groups that were capable of synergistically binding metal cations. In this way, the adsorption of europium(III) on modified silica gel was improved [55]. 

In terms of lanthanides and trivalent actinides, Fan et al. investigated the adsorption performance and mechanism of a novel porous BCPDTPA/SiO_2_-P extraction resin for trivalent actinides (Am) and lanthanides (Eu). The results showed that the affinity of the BCPDTPA/SiO_2_-P resin for Am(III) was higher than that of Eu(III) within the range of experimental conditions [56]. Artiushenko et al. used a silica-based adsorbent immobilized with aminobis (methylenephosphonic acid) fragments in a dispersive solid-phase extraction mode for the selective recovery of rare-earth elements from acidic solutions. Under acidic conditions, 14 rare-earth elements were completely absorbed in less than 5 min. This method resulted in a 100-fold increase in the amount of rare-earth elements extracted from the leachate [57]. The above studies have shown that this type of adsorbent has good adsorption capacity with high recovery and high stability under acidic conditions and is effective for the desorption of metals and recovery of adsorbent.

#### 2.2.2. Silica-Based Mesoporous Materials Containing Amino Groups

Am-SiO_2_ cannot be used to separate rare-earth elements from metal-containing solutions due to lack of selectivity, and adsorbents with immobilized oxygen-containing chelating ligands will show better efficiency in recovering rare-earth elements [57]. Hu Y et al. obtained a new silica-based adsorbent material by grafting a series of synthesized preorganized bidentate phthaloyl diamide (PA) ligands onto macroporous three-dimensional (3-D) KIT-6 mesoporous silica. The grafted PA-type ligands significantly improved the extraction performance of the adsorbent for REEs compared with the homogeneous analogs [58]. Hu and colleagues grafted a series of tetravalent phenyl dioxydiamine (PDDA) ligands on the surface of KIT-6 silica gel and selectively separated rare-earth ions by adjusting the occlusion angle of the chelating ligands [59]. Ravi et al. obtained mesoporous silica with accessible adsorption sites and good stability by grafting ethylenediaminetetraacetic acid (EDTA) on two representative mesoporous silica (KIT-6 and KCC-1). The results showed that it has a large adsorption capacity for Nd(III), with maximum adsorption capacities of up to 109.8 and 96.5 mg/g, respectively [60]. Huang Y et al. successfully synthesized lanthanide ion-imprinted polymer (La-IIP/SBA-15/Y) using the surface ion-imprinting method. The results showed that La-IIP/SBA-15/Y had good selectivity and good experimental elution and regeneration performance. The best elution effect was 2 mol/L of hydrochloric acid [61].

Novel amino-functionalized mesoporous zirconia-silica (ZNSi) composites were prepared and used for the adsorption and in situ immobilization of simulated Nd in aqueous solution by Chen et al. The Nd adsorption capacity of the obtained composites was up to 31.14 mg/g [62]. Lakić et al. grafted diamino functional ligands grafted via alkyne linkers on the surface of dense silica nanoparticles as a way to improve the selectivity for LTM. The metal coordination ratio of the prepared organosilica materials to Ni cations was 1:1 [63]. Artiushenko et al. were able to quantitatively pre-concentrate a constant amount of REEs from contaminated water with pH ≥ 3 on a silica-based adsorbent with covalently immobilized amino-bis (methylenephosphonic acid) (SiO_2_-AdPA). The complete desorption of all REEs was achieved using a 10-2 mol/L EDTA solution (pH = 8.0), whereas the acidic treatment required 1.0 mol/L of HNO_3_ [64]. The above studies have shown that this type of adsorbent is completely stable in at least three consecutive adsorption/desorption cycles, with no degradation of its adsorption properties, good reusability, and better affinity for rare-earth elements than the bulk silica with different functional groups. The adsorption properties are shown in Table 4.

#### 2.2.3. Silica-Based Mesoporous Materials Containing Carboxyl Groups

Florek et al. modified a mesoporous KIT-6 silica material with a diethylene glycolamide (DGA)-type ligand and successfully achieved the highly selective separation of lanthanide rare-earth elements [65]. Three identical DGA-based hybrid silica adsorbents were further synthesized in their subsequent work and applied to rare earth extraction and purification [66]. Cui J et al. also prepared a DGA-modified silica adsorbent and used it for the selective adsorption of Dy(III) [67]. Cui J among others used APTES and DTPADA for the two-step modification of SBA-15 derived from fly ash to obtain DTPADA-SBA-15 adsorbent [68]. The above studies have shown that the adsorbents have excellent selective adsorption performance for REEs such as Eu, Gd, Tb, Nd, and Sm in acidic solutions with a variety of competing ions, and they can be recycled many times without the depletion of the adsorption performance.

#### 2.2.4. Other Silicon–Based Mesoporous Materials

Iftekhar et al. studied and synthesized two different acid-modified cellulose-based silica (CLx/SiO_2_) nanocomposites. The experimental results showed that the CLx/SiO_2_ composites prepared through sulfuric acid modification were more favorable for adsorption [69]. Daulay et al. prepared Na-A zeolite from fly ash (CFA) and used it for the adsorption of Ce(III). The experimental results showed that the desorption efficiency was 97.22% for four cycles. Adsorption tests using real wastewater samples showed an adsorption efficiency of 83.35% [70]. Ramasamy et al. prepared activated carbon (AC) and silica composites via the tuned hybridization and ligand grafting methods, which showed that hybridization of carbon-based and silica-based materials not only preserves the adsorption properties of silica-based materials but also improves the stability of the materials under acidic conditions [71]. Shahed V G et al. prepared Ni/SBA-15-promoted catalysts with different Sm promoter dosages (0.5, 1.5, 3, and 6 wt.%). The addition of Sm promoter resulted in a significant increase in the catalytic activity, with the catalyst containing 3 wt.% of Sm giving the highest H_2_ yield of about 66% [72].

The above studies show that functionalized silica-based adsorbent materials containing Lewis base (N, P, –COO, etc.) groups can effectively achieve rare earth separation under certain conditions, giving them good adsorption selectivity and reusability, and improving adsorption efficiency and regeneration performance.

### 2.3. Porous Organic Polymer Materials

Porous organic polymers(POPs) are highly suitable materials for various chemical processes; the adsorption properties of porous structures and polymers are influenced by their morphology. POPs are considered strong contenders for applications such as gas adsorption, drug delivery, sensing, organic pollutant removal, energy storage, and catalysis. POPs exhibit exceptional characteristics, such as a well-organized structure, large specific surface area, adjustability and customizability, frameworks without metal components, and outstanding physicochemical stability. By incorporating diverse ion exchange and chelating groups into porous organic polymers, their specificity for capturing certain substances is significantly improved. This modification results in a more effective adsorbent that offers several advantages, including a straightforward adsorption process, minimal energy usage, rapid adsorption rate, and convenient recovery and reuse for multiple cycles.

#### 2.3.1. Porous Polymers Based on Chitosan and Cyclodextrins

Chitosan is the second most abundant biopolymer and is the preferred adsorbent due to its non–toxicity, biodegradability, anti-microbial, and excellent physicochemical properties [73]. Chitosan has received attention in many research fields due to its ion exchangeability, excellent surface area, chemical bonding, chiral, biodegradability, hydrophilicity, and biocompatibility, which make chitosan an ideal material.

In some studies, Ren et al. designed a chitosan-based Y(III) imprinted (CYIT) hydrogel with reversible thermo-responsiveness, whose maximum adsorption capacity for Y(III) could reach up to 160.0 mg/g, which was much higher than the corresponding performance of similar hydrogels, with high selectivity and reproducible performance [74]. Zhang et al. synthesized a high-performance aerogel composite, MWCNT-PDA-CS-GO, by employing a sequential method that involved heat and mass transfer under low-temperature and low-pressure conditions [75]. The experimental findings demonstrated that the MWCNT-PDA-CS-GO aerogel achieved a maximum adsorption capacity of 150.86 mg/g for Gd(III) at a pH of 7.0. Wang et al. utilized the oxygen–phosphorus functional group in sodium tripolyphosphate, which combined it with chitosan [76]. It possesses excellent thermal stability and can be easily separated using magnetic properties. Research has found that the coordination effect of surface functional groups and the special ion-imprinted pore structure contribute to the high selectivity of IIP-BT/CoFe_2_O_4_@SiO_2_ for Sc(III).

**Figure 6:**
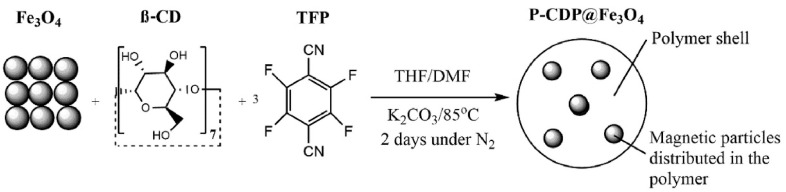
Schematic representation of the synthesis of P-CDP@Fe_3_O_4_ [77].

As Figure 6 shows, Nkinahamira et al. created a magnetic composite of porous β-cyclodextrin polymer (P-CDP@Fe_3_O_4_) that rapidly and selectively recovers rare-earth elements (Nd, Gd, Eu, and Y) from industrial wastewater, achieving adsorption capacities of up to 9.59 mg/g at 25 °C and reaching equilibrium in less than 10 min [77]. The P-CDP@Fe_3_O_4_ addresses the clogging and high backpressure issues associated with P-CDP by being easily separable with an external magnet, and it demonstrates excellent selectivity with recovery efficiencies for REEs ranging from 62% to 100% in both model studies and industrial wastewater. This material efficiently adsorbed rare-earth elements Nd, Gd, Eu, and Y within a short period of 10 min at a temperature of 25 °C. When the initial concentration was 100 mg/L, the maximum adsorption capacity ranged from 7.76 to 9.59 mg/g. Furthermore, Nkinahamira’s research team also produced a composite material called PCDP–M–SHM in order to improve the adsorption of rare earths with P-CDP@ Fe_3_O_4_. PCDP–M–SHM demonstrated the ability to achieve adsorption equilibrium in just 300 min [78]. It also exhibited superior adsorption capabilities for rare-earth elements, with maximum adsorption capacities of 64.28, 49.24, 51.16, and 40.92 mg/g for the rare-earth elements Y, Nd, Eu, and Gd, respectively, at a temperature of 25 °C. Jemli et al. prepared green, inexpensive, and easily synthesized β-cyclodextrin nanosponges for the effective adsorption of cerium and lanthanum from aqueous solution [79]. The adsorption capacity of the material was 625.34 and 773.29 mg/g for Ce and La, respectively.

Biopolymers have been widely used and researched as potential materials because they are widely available, inexpensive, biodegradable, and non-toxic. Due to these properties, recent research and development have focused on the use of these materials as potential adsorbents with significant adsorption effects.

#### 2.3.2. Porous Polymers Based on Ion Imprinting

Ion imprinting technology is a favorable functionalization of adsorption technology, which makes the adsorbent material have a high affinity and selectivity for the target ions. As an important branch of molecular imprinting technology, ion imprinting technology has many advantages due to its coordination interactions, such as pre-designed specificity, stability, and specific recognition. The polymers obtained through ion imprinting not only have good structural stability but also feature fast adsorption rates and high adsorption selectivity. These benefits have made ion-imprinted porous polymers widely noticed in the field of rare-earth ion adsorption and separation [80].

Chen et al. fabricated a new polymer-encapsulated membrane (PIM) by incorporating poly(vinyl alcohol)-ethylene copolymer (EVOH) as a hydrophilic additive [81]. The calculated separation factor reached a remarkable value of 3.78, significantly surpassing the results obtained from previous studies on both solvent extraction and membrane separation. Yang et al. suggested a method that combines surface imprinting and defect engineering strategies to create Gd(III) ion-imprinted polymers (G-IIPs) using hybrid-conjugated Zr-MOFs [82]. The findings indicated that the G-IIP-3, which was prepared with a significant number of defects, exhibited a substantial adsorption capacity of 181.75 mg/g for Gd(III) and a rapid equilibration time of 30 min; Xu et al. synthesized a novel O-group-modified porous coordination polymer (CP) with good adsorption capacity for rare-earth ions, and the results showed that the adsorbent had good adsorption performance for low concentrations of rare earth ions (1 ppm), which was reached equilibrium in only 10 min and had good selectivity in the presence of competing ions [83].

**Figure 7:**
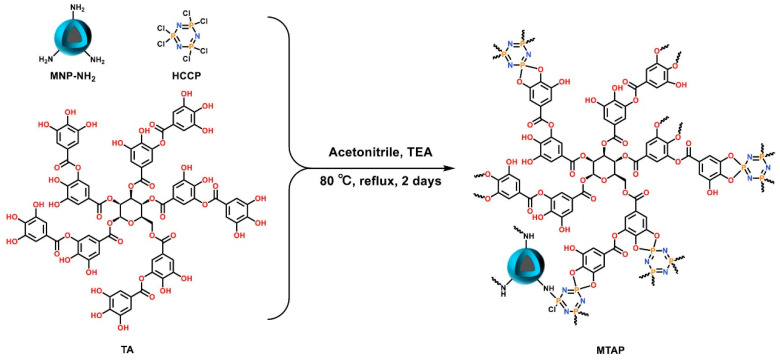
The synthesis of MTAP [86].

Zheng et al. introduced a method called double template-docked ion-imprinted (DTD-OII) per layer [84]. They successfully created double template-docked oriented ion-imprinted mesoporous bilayer films (IIBFs) for the purpose of selectively adsorbing Nd(III) and Dy(III). In comparison to other adsorbents for rare-earth elements, the IIBFs demonstrated rapid equilibration and high adsorption capacity in acidic solutions. Han et al. synthesized copolymers through a reversible addition-breakage chain transfer reaction, utilizing styrene (S) and glycidyl methacrylate-iminodiacetic acid (GMA-IDA) as monomers [85]. The experimental results indicate that HCP-2 exhibits a maximum adsorption capacity of 145.65 mg/g for terbium at pH 5.8 and a temperature of 323 K, demonstrating notable competitiveness compared to other reported porous adsorbents. Liu et al. fabricated a magnetic porous organic polymer, MTAP (Figure 7), utilizing natural polyphenolic tannins as monomers and hexachlorocy-clotriphosphononitrile as a cross-linking agent. The experimental findings indicate that the adsorption of rare-earth elements, specifically Ce, Nd, Eu, and Gd, occurs rapidly, with adsorption equilibrium being achieved within 2 min [86].

#### 2.3.3. Porous Polymers Based on Phosphorylation

Phosphate-rich adsorbent materials are able to achieve higher REE adsorption capacity and maintain high chemical stability after multiple acid regenerations. In addition, the formation constants of phosphates with other common trivalent cations such as Fe(III) and Al(III) are lower than lanthanides, which can provide greater selectivity for RE than these other competing cations in solution.

**Figure 8:**
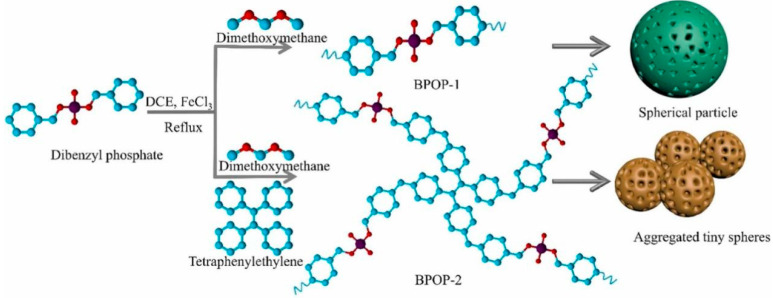
Schematic syntheses of phosphate-based porous organic polymers BPOP-1 and BPOP-2 [92].

In some studies, Archer et al. synthesized a rare-earth chelated polymer resin containing a phosphonate ester, which has a higher REE capacity relative to surface-functionalized solid-phase extractants and uses covalently linked ligands to confer higher chemical stability over multiple cycles of acid regeneration and reuse [87]. Ni et al. synthesized a novel lanthanum(III) molecularly imprinted polymer (La-IIP) using vinylphosphonic acid (VPA) as a functional monomer [88]. The adsorption capacity of La-IIP was 62.8 mg g^−1^, and the selectivity coefficient, kLa/Cu, was 54.57. Zhang et al. developed a phosphate polymer nanogel (PPN), with high phosphorus content and abundant coordination sites, PPN exhibits high selectivity and adsorption capacity for REE, even in the presence of a large number of competing ions [89]. Rodrigues et al. designed a carbamoylmethylphosphonated water-soluble polymer with two water-soluble polymeric adsorbents bearing carbamomoylmethylphosphonate fractions (P(CPAAm 6C) and hP(CPAAm 6C)) [90]. In the case of P (CPAAm 6C), adsorption experiments proved that Q_max_(Th) should be higher than 1.5 mmol/g. Adsorption with hP (CPAAm 6C) showed that Q_max_(U) should be greater than 2.75 mmol/g. Liang et al. synthesized a rare-earth adsorbent material, MIL-101(Cr)-SMA-ED-PMG, with abundant carboxyl and phosphoric acid Lewis hard base groups using polymer chain Cr-MOF for modification [91]. The maximum adsorption of Nd (III) and Eu (III) was 102.7 mg/g and 110.4 mg/g, respectively, at the optimum pH of 5. Ravi et al. incorporated phosphate functional groups into POP materials to achieve materials with elevated specific surface area and pore volume (Figure 8). The experimental findings demonstrated the preferential capture of Dy(III) ions by the BPOP material in comparison to other competing cations, including Zn(II), Cu(II), Mg(II), and K(I) [92]. 

The above research experiments show that porous polymers enriched with phosphoric acid have better selectivity and adsorption of rare-earth elements, and their reusability is good, so the solid-phase extraction material with a high density of phosphoric acid groups is an ideal material for the recovery of rare earths. A comparison of adsorption is shown in Table 5.

### 2.4. Metal–Organic Framework (MOF) Materials

#### 2.4.1. MOF Material Profile

MOFs represent an innovative class of crystalline porous materials, composed of metal ions or metal clusters (acting as nodes) and organic ligands (serving as linkers) interconnected through coordination bonds. They have attracted much attention in coordination chemistry in recent years. MOFs offer advantages such as high porosity, low density, a large specific surface area, tunable pore size, varied topology, and customizability, and have been widely used in the fields of rare earth recovery. However, most of the MOF materials have poor stability under acidic conditions, which greatly limits their practical applications.

#### 2.4.2. Rare Earth Separation with MOFs

Lee et al. used organic functional groups such as -NH_2_, -ED, -DETA, and -PMIDA to post-modify Cr-MIL-101 and found that these functionalized MOFs can efficiently adsorb, separate, and recover rare-earth elements (REEs) from aqueous solutions. In addition, they found that MIL modified with N-(phosphonomethyl)iminodiacetic acid (PMIDA) had better adsorption capacity for La, Ce, Nd, Sm, and Gd than other organic groups [93]. Ryu et al. investigated the adsorption performance of MIL-101-PMIDA on lutetium (Lu) and yttrium (Y). They found that the adsorption capacity of MIL-101-PMIDA for Lu and Y was 63.4 mg/g and 25.3 mg/g, which were 3.7 and 1.4 times higher than that of PMIDA-SBA-15, respectively, at a pH of 5.0 ± 0.2. and 1.4 times that of PMIDA-SBA-15, respectively [94]. Fonseka et al. prepared a series of Cr-MIL for the selective recovery of europium (Eu) from zinc ore leach solution through the in situ grafting of functional groups on chromium sites. It was found that the maximum Eu adsorption capacity of Cr-MIL-PMIDA modified by PMIDA was 69.14 mg/g at pH 5.5 [95]. Liang et al. obtained MIL-101(Cr)-SMA-ED-PMG by introducing abundant carboxyl and phosphoryl functional groups to Cr-MIL. At the optimal pH = 5, the maximum adsorption capacity of MIL-101(Cr)-SMA-ED-PMG for Nd^3+^ and Eu^3+^ was 102.7 mg/g and 110.4 mg/g, respectively. They demonstrated that the coordination between phosphate groups and rare-earth ions is the main driving force in the adsorption process and that carboxyl groups and nitrogen-containing functional groups also contribute to the adsorption effect to a certain extent, by means of characterization such as XPS [91]. 

**Figure 9:**
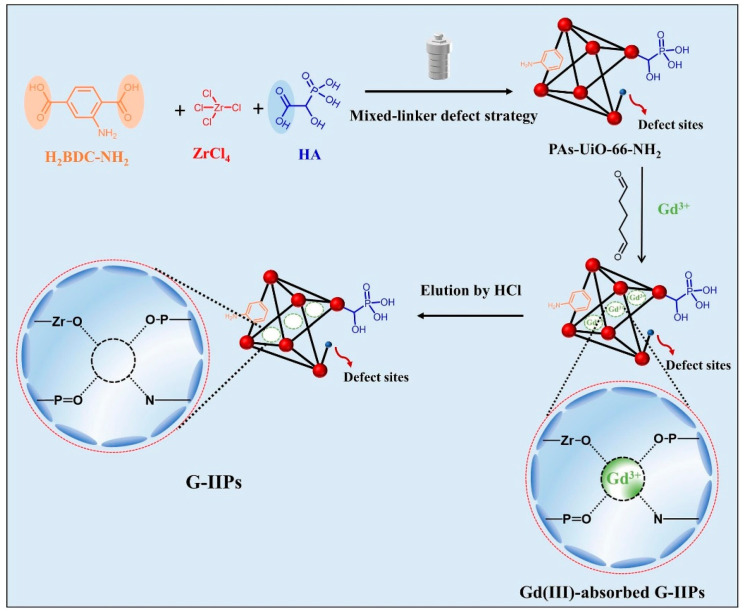
Schematic illustration of PAs-UiO-66-NH_2_ and G-IIPs [82].

Yang et al. prepared ion-imprinted MOFs (G-IIP-3) with rich defects and a high specific surface area through surface imprinting and defect engineering strategies (the synthesis schematic is shown in Figure 9). G-IIP-3 exhibits high adsorption capacity for Gd(III) (181.75 mg/g) and a short equilibrium time of 30 min, while still maintaining high selectivity even in the presence of interfering ions [82]. Zhao et al. used a certain amount of ZrOCl_2_-8H_2_O, H_1_ (1,2,4-benzenetricarboxylic acid), and H_2_ (1,2,4,5-benzenetetracarboxylic acid) mixed at room temperature to obtain nanoporous metal–organic frameworks (UiO-66-H_1_/H_2_-a) with a high adsorption capacity of up to 150–250 mg/g for a wide range of rare-earth ions (including Sm (III), Eu (III), Gd (III), Tb (III), Dy (III), Ho (III), Er (III), Tm (III), and Yb (III)) [96]. Zr-O clusters all play a key role in the adsorption process of rare earths, which provides a new research idea for the application of Zr-O clusters in the synthesis of adsorbent materials.

**Figure 10:**
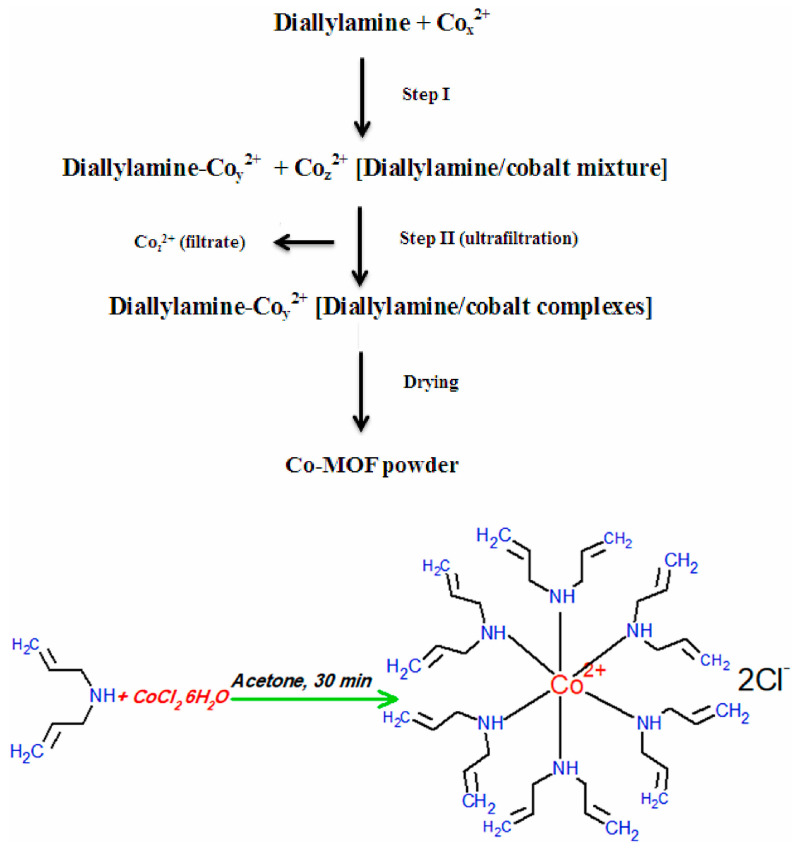
The provided text describes a simplified flow diagram illustrating the synthesis procedures of Co−MOF using the sol–gel method [97].

**Figure 11:**
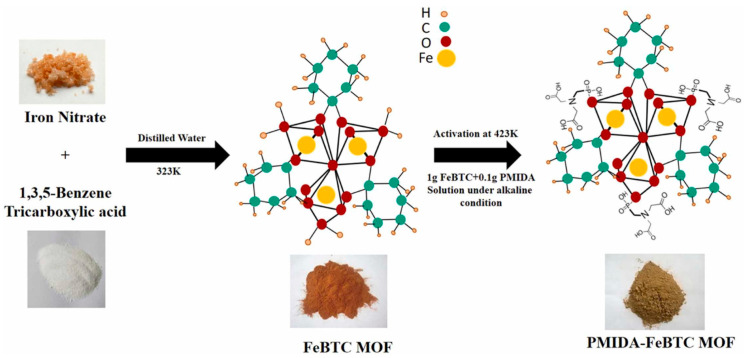
Scheme for synthesis of PMIDA-functionalized FeBTC MOF [99].

Khalil et al. used cobalt chloride, diallylamine, and methanol to form a metal–organic structure through self-assembly to obtain Co-MOF (the synthesis schematic is shown in Figure 10); the adsorption capacity of Co-MOF for Ce(III) was found to be 74.65 mg/g at pH 5.1 [97]. Song et al. self-assembled with N-heterocyclic imide ligand, homophthalic acid diimide, and cadmium nitrate tetrahydrate to form amino-functionalized microporous two-dimensional MOFs (Cd-PDI-2D) and three-dimensional MOFs (Cd-PDI-3D) at 115 °C and 85 °C, and Cd-PDI-2D showed excellent adsorption of all Ln(III) at pH = 6.8. In contrast, almost no adsorption of Cd-PDI-3D Ln(III) was observed [98]. Sinha et al. utilized N-(phosphonomethyl)iminodiacetic acid (PMIDA) coordinated at the Fe site of FeBTC to produce PMIDA@FeBTC MOF-functionalized metal–organic skeleton material through complexation (the synthesis schematic is shown in Figure 11), which adsorbed as high as 232.5 mg/g at 298K [99]. Allahyari Sareh Ammari et al. used a mixture of Zn(OAc)_2_·3H_2_O, 1,4-benzene dicarboxylic acid (H_2_bdc), benzimidazole (bim), NaOH, etc., stirred in an autoclave for 72 h to obtain [Zn(bim)_2_(BDC)]_n_ metal–organic frameworks (ZBB), which at pH = 7, showed a La(III) adsorption amounting to 156.7 mg/g [100]. Paz Roxana et al. used Zn(NO_3_)_2_·6H_2_O and terephthalic acid (H_2_BDC) dissolved in N,N-dimethylformamide (DMF), among other materials, and by heating, cooling, separating, and drying, obtained (Zn-BDC) MOF, which adsorbed up to 598.0 mg/g of Eu(III) at pH = 7 [101]. Many of the above studies were only carried out under weakly acidic conditions (pH of about 7), and when the pH is too low and the solution conditions are harsh, their structures will be difficult to preserve, greatly limiting their applications. In the future, their strength in aqueous solution can be improved by increasing the strength of the surface coordination bonds.

Li et al. used hydrothermal and impregnation methods to combine polyethyleneimine (PEI) with LaBDC to form a novel LaBDC@xPEI composite, which was enriched with a large number of amine, carboxyl, and hydroxyl groups on the surface, which had a high affinity for Gd(III). at pH = 5.5; LaBDC@50% PEI with different PEI loadings had the highest lanthanide MOFs had the highest adsorption (181.77 mg/g), which was about 5.1 times that of LaBDC [102]. And, Li et al. also successfully added 4,6-diamino-2-mercaptopyrimidine (DMP) to LaBDC using lanthanum as the core metal through impregnation with benzene dicarboxylic acid, and the adsorption capacity of LaBDC@60% DMP for Gd(III) could reach 241.4 mg/g, which is about 6.8 times higher than that of LaBDC, at 25 °C, pH = 5.5, and 60% DMP. LaBDC@50%PE and LaBDC@60%DMP are promising for the adsorptive removal of Gd(III) for the effective separation and recovery of wastewater [103].

In Chen et al.’s study, two-dimensional MOFs and GO nanocomposites (2D Zn-BDC MOF/GO) were synthesized using a solvent–thermal interlayer confinement strategy, with a maximum adsorption of 344.48 mg/g of rare-earth elements (Sc, Y, La, Ce, Pr, Nd, Eu, Gd, Dy, Er, and Tm) at pH ≈ 4. This work provides a new direction for the selective separation of rare earths [104].

As can be seen from Table 6, organometallic framework materials have great adsorption potential for rare earth recovery(), but the relatively poor water and chemical stability of organometallic framework materials and the inability to maintain their structure under harsh conditions greatly limit their practical application.

## 3. Conclusions

This paper reviews recent research reports on the performance of porous carbon-based, porous silicon-based, porous organic polymers, and porous metal–organic frameworks (MOFs) for the adsorption of rare-earth elements, and by comparing the results of this research, it can be found that each of these materials has its own advantages and disadvantages. The cost of adsorbents and green renewable and excellent adsorption properties are the main challenges to face in order to realize the industrial application of the adsorption method in the field of rare earth recovery. Therefore, among the many adsorbent materials, we believe that carbon-based and silicon-based adsorbents have the greatest potential for application in the adsorptive recovery of REEs from mine leachate and wastewater. However, carbon- and silicon-based adsorbents require further modification to achieve better adsorption performance due to the limited content of functional groups indicated by themselves.

Due to the fact that the phosphoric acid group can still maintain excellent rare-earth coordination performance under acidic conditions, future research is recommended to focus on the modification methods of the phosphoric acid group on carbon-based and silicon-based porous materials, in order to further enhance the adsorption performance of carbon-based and silicon-based adsorbents. With the continuous deepening of the research, the technology of using porous materials to adsorb rare-earth elements will be further improved, and the prospect of industrial application will be broader.

## Figures and Tables

**Table 1:** Preparation method and adsorption properties of activated carbon.

Carbon Source	Preparation and Modification Method	pH	Adsorption Capacity (mg/g)	Detection Methods	References
Navel orange peel	H_3_PO_4_ activation Functionalized with glycine.	7	Gd(III) = 48.5	ICP-OES	[27]
Soybean pod	High-temperature pyrolysis ZnCl_2_ activation	2 6	Ce(III) = 107.7 La(III) = 127.2	ICP-OES	[28]
Grape pruning waste	High-temperature pyrolysis ZnCl_2_ activation	4–6	Ce(III) = 48.45 La(III) = 53.65	ICP-OES	[29]
Palm leaves	HNO_3_ activation	3	Eu (III) = 810	Arsenazo (III) method	[35]
Solidago canadensis	Co-precipitation High-temperature pyrolysis Composite with other materials	6	Eu(III) = 714	Ultraviolet spectrophotometry	[31]

**Table 2:** Preparation method and adsorption properties of graphene oxide.

GO-PER	Hydrothermal-assisted assembly Freeze-dried	5.5	La(III) = 27.92 Y(III) = 30.44 Nd(III) = 43.2 Er(III) = 48.08 Yb(III) = 52.4	ICP-OES	[41]
GO-CZ	Hydrothermal self-assembly	4.0–5.0	Y(III) = 14.2 Nd(III) = 9.68	ICP-OES	[42]
GO-ABT	Hydrothermal self-assembly 2-Aminobenzothiazole	4.5	La(III) = 23.36 Er(III) = 32.23 Yb(III) = 30.95 Nd(III) = 30.92 Y(III) = 12.97 Eu(III) = 42.53	ICP-OES	[43]
GO-ZG	Hydrothermal self-assembly N-Benzoxycarbonyl glycine	5.27	La(III) = 44.56 Er(III) = 50.48 Yb(III) = 53.64 Nd(III) = 45.96 Y(III) = 30.63	ICP-OES	[44]
GO-Bicine	Covalent coupling N,N-Bis(2-hydroxyethyl) glycine	4.5	Eu(III) = 3.62 Nd(III) = 5.09 Y(III) = 24.40	ICP-OES	[45]
GO-BT	Covalent grafting Bis(2-hydroxyethyl)amino-2-(hydroxymethyl)−1,3-propanediol	Unknown	Y(III) = 24.89 Yb(III) = 28.13 Eu(III) = 18.77 Sm(III) = 57.46 Nd(III) = 26.25 La(III) = 33.12 Er(III) = 22.64 Pr(III) = 6.529	ICP-OES	[46]
GO-APTS	Covalent grafting 3-[2-(2-aminoethylamino) ethylamino]propyl-trimethoxysilane	6.0	Er(III) = 93.4 Eu(III) = 103.2 Lu(III) = 83.7 Tm(III) = 97.2 Y(III) = 48.3 Yb(III) = 92.8 Ho(III) = 110.0	ICP-OES	[47]

**Table 3:** Modification methods and rare earth adsorption properties of CNTs.

Adsorbent	Preparation and Modification Method	pH	Adsorption Capacity (mg/g)	Detection Methods	References
MWCNT@Fe_3_O_4_	Fe_3_O_4_ wrap	Unknown	La(III) = 23.23	ICP-AES HPLC	[48]
COOH-CNTs/CS-IIS	Graft modification	7.0	Gd(III) = 71.95	ICP-AES	[49]
CNT-FFA-PAA	Graft modification	6.0	Eu(III) = 130.8	Unknown	[50]
CT-CNTs SA-CNTs	Purchased	9.37	REE mass >90%	Neutron Activation Analysis	[51]

**Table 4:** Comparison of adsorption properties of aminosilica-based mesoporous materials.

Ligand	Preparation and Modification Method	pH	Adsorption Capacity (mg/g)	Detection Methods	References
KIT-6-1,2-PDDA KIT-6-1,3-PDDA	Group graft	6.33 5.0	Lu(III) = 19.8 Ce(III) = 12.5	ICP-MS ICP-MS	[58]
DTPADA PAA	Group graft	2.06	Nd(III) > Gd(III) > Ho(III)	ICP-MS	[59]
EDTA	Group graft	6.0	Nd(III) = 109.8	ICP-AAS	[60]
0.2ZNSi	APTES modification	6.0	Nd(III) = 31.6	ICP-OES	[62]

**Table 5:** Comparison of adsorption properties of phosphorylated porous polymer.

Ions	Phosphorylated Porous Polymers	pH	Adsorption Capacity (mg/g)	Detection Methods	References
REEs	Rare-earth chelate polymer resins	Unknown	unknown	ICP-MS	[87]
La(III)	Phosphonic-based ion-imprinted polymer	3.0–7.0	62.8	Unknown	[88]
Nd, Gd, Ho (III)	Phosphate polymer nanogels	7.0	Nd = 311 ± 28 Gd = 316 ± 38 Ho = 249 ± 29	ICP-MS	[89]
Gd(III)/Th(IV)/U(VI)	Carbamoylmethylphosphonated water-soluble polymers	1.0	P(CPAAm 6C): >1.5 mmol/g HP(CPAAm 6C): >2.75 mmol/g	ICP-OES	[90]
Nd(III) Eu(III)	MIL-101(Cr)-SMA-ED-PMG	5.0	102.7 110.4	ICP-OES	[91]

**Table 6:** Preparation method and adsorption properties of MOFs.

Adsorbent	Preparation and Modification Method	pH	Adsorption Capacity (mg/g)	Detection Methods	References
G-IIP-3	Mediating ligand	unknown	Gd(III) = 181.75	ICP-OES	[82]
MIL-101(Cr)-SMA-ED-PMG	Condensation reaction	5.0	Nd(III) = 102.7 Eu(III) = 110.4	ICP-OES	[91]
MIL-101-PMIDA	PMIDA-modified Cr-MIL-NH_2_	5.0 ± 0.2	Lu(III) = 63.4 Sc = 25.3	ICP-MS	[94]
Cr-MIL-PMIDA	Hydrothermal synthesis	5.5	Eu(III) = 69.14	ICP-MS	[95]
UiO-66-H_1_/H_2_-a	Self-organization	unknown	Sm(III)Eu(III)Gd(III) Tb(III) Dy(III)Ho(III)Er(III)Tm(III)Yb(III) = 150~250	ICP-OES	[96]
Co-MOF	Self-organization	5.1	Ce(III) = 74.65	UV–visible spectrophotometer	[97]
PMIDA@FeBTC MOF	Complexation reaction	unknown	La(III) = 232.5	ICP-AES	[99]
[Zn(bim)_2_(BDC)]_n_	High-temperature self-assembly	7	La(III) = 156.7	ICP-AES	[100]
(Zn-BDC) MOF	High-temperature self-assembly	7	Eu(III) = 598.0	ICP-AES	[101]
LaBDC@50%PEI	Maceration	5.5	Gd(III) = 181.77	ICP-AES	[102]
LaBDC@60%DMP	Maceration	5.5	Gd(III) = 241.4	ICP-AES	[103]
2D Zn-BDC MOF/GO	Dissolution Thermal Interlayer Closure Strategy	4	(Sc, Y, La, Ce, Pr, Nd, Eu, Gd, Dy, Er, Tm) = 344.48	ICP-AES	[104]
